# Mental health and social support among glaucoma patients enrolled in the NIH *All of Us* COVID-19 Participant Experience (COPE) survey

**DOI:** 10.1186/s12886-023-02771-1

**Published:** 2023-02-13

**Authors:** Arash Delavar, Jennifer J. Bu, Bharanidharan Radha Saseendrakumar, Robert N. Weinreb, Sally L. Baxter

**Affiliations:** 1 Division of Biomedical Informatics, Department of Medicine, University of California San Diego, La Jolla, CA USA; 2 Division of Ophthalmology Informatics and Data Science, Viterbi Family Department of Ophthalmology and Shiley Eye Institute, University of California San Diego, 9415 Campus Point Drive MC 0946, La Jolla, CA 92093 USA

**Keywords:** COVID-19, glaucoma, Depression, Mental health, Social support

## Abstract

**Background:**

The COVID-19 pandemic created many challenges for our society. In this study, we explore how measures of mental health, coping strategies, and social support during the pandemic varied by glaucoma status.

**Methods:**

A cohort of patients aged 40 and over enrolled in the NIH *All of Us* Research Program, a nationwide longitudinal cohort, who answered the COVID-19 Participant Experience (COPE) survey was obtained. We analyzed several measures of mental health, coping strategies, and social support used during the early stages of the COVID-19 pandemic. Surveys were recurring and answered from May 2020 to February 2021. Demographics and the most recently answered survey responses were obtained and stratified by glaucoma status. Pearson’s Chi-squared tests and multivariable logistic regressions adjusting for age, gender, race, ethnicity, and income were used to generate *p*-values, odds ratios (ORs) and 95% confidence intervals (CIs) between outcome measures and glaucoma status.

**Results:**

Of 42,484 patients who responded to *All of Us* COPE survey items, 2912 (6.9%) had a diagnosis of glaucoma. On Pearson’s Chi-squared tests glaucoma patients were less likely to report drinking alcohol (*P* = 0.003), eating more food than usual (*P* = 0.004), and using marijuana (*P* = 0.006) to cope with social distancing than those without a diagnosis of glaucoma. Further, glaucoma patients had lower rates of probable mild, moderate, or severe depression as calculated by Patient Health Questionnaire-9 (PHQ-9) scores (*P* < 0.001) and had lower rates of reporting some or a lot of stress from social distancing (*P* < 0.001). However, glaucoma patients were less likely to report having someone to help prepare meals (*P* = 0.005) or help with daily chores (*P* = 0.003) if they became sick with COVID-19. In multivariable logistic regression analyses adjusting for confounding factors, no differences were found for measures of mental health or social support.

**Conclusions:**

Glaucoma patients did not fare worse on many measures of mental health and coping strategies during the early stages of the COVID-19 pandemic compared those without glaucoma. However, a substantial proportion of glaucoma patients still endorsed stress, social isolation, and probable depression, representing challenges for disease management.

## Background

The COVID-19 pandemic and its associated lockdowns created unprecedented challenges for our society [1]. Though social distancing, remote work/school, and closure of many public and private spaces undoubtedly saved lives, the mental health burden of these interventions is thought to be substantial [1]. A number of studies have demonstrated the mental health burden of the pandemic and lockdowns on the worldwide population, with one meta-analysis of international studies showing a pooled prevalence of depression of 25% after the pandemic compared to a global estimate of 3.44% in 2017 [2]. Another meta-analysis estimated an additional 53.2 million cases of depression worldwide attributable to the pandemic [3]. Further, a US-based study found 3-fold greater prevalence of depressive symptoms during the pandemic than before [4].

The pandemic impacted many fields of medicine, ophthalmology included [5]. The impacts of office closures and delays in care such as postponing clinic follow-up, medication refills, and surgery have not been fully revealed [6, 7]. This pandemic has proven stressful for ophthalmology patients as well, with one study finding a majority of patients were concerned about limitations in healthcare access and were fearful of disease progression [8]. This may be especially impactful for those with chronic conditions that affect the eye.

Glaucoma is a progressive neuropathy of the optic nerve and is the leading cause of blindness worldwide [9]. Risk factors include age, race/ethnicity, and family history, but the main modifiable risk factor for disease progression is intraocular pressure [10]. However, proper management requires close monitoring of optic nerve health and may include complicated medical regimens, which may be more challenging for those with mental health conditions to manage [11]. Further, glaucoma patients are thought to be especially vulnerable considering some studies have shown they have higher rates of anxiety and depression than the general population [12] – with risk factors including increasing age and glaucoma severity [13, 14] – as well as lower incomes on average [15].

There is evidence that the COVID-19 pandemic decreased quality of life metrics among those with eye disease, including increased fear about losing vision [16, 17]. Further, the pandemic is thought to have increased the prevalence of anxiety which may have had a negative effect on treatment adherence among glaucoma patients in Croatia [18]. However, the extent to which the pandemic may have affected other factors of mental health among glaucoma patients is not well characterized. In this study, we leveraged a nationwide survey to characterize experiences during the COVID-19 pandemic, including measures of probable depression, coping strategies, and social support, among both glaucoma and non-glaucoma patients in the United States.

## Methods

### Study population

We obtained data from the National Institutes of Health (NIH) *All of Us* Research Program, a nationwide database with an emphasis on diversity, aiming to enroll at least 1 million people [19]. At the time of our analysis in February 2022, 331,360 participants had enrolled. Institutional Review Board (IRB)/Ethics Committee approval was obtained. Participants provided written informed consent at enrollment in the study, which was approved by the NIH *All of Us* IRB. *All of Us* collects a wide range of data from participants, including physical measurements, electronic health record (EHR) data, survey data, wearable data, and biospecimen collection [19]. *All of Us* data undergo de-identification processes prior to becoming available to researchers [19]. Secondary analyses of de-identified data, such as those evaluated for our study, are considered non-human subjects research, which was verified by the University of California San Diego (UCSD) IRB. The study adhered to the tenets of the Declaration of Helsinki. Per the *All of Us* Research Program data sharing policies, cells with less than 20 respondents are suppressed.

**Fig. 1:**
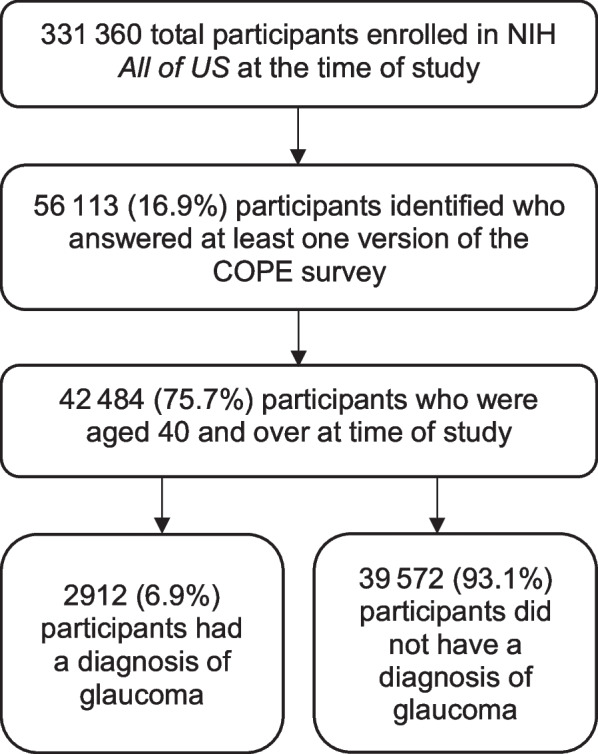
Flowchart of exclusion criteria leading to a final study population of 42,484 patients 40 years and older enrolled in the NIH *All of Us* Research Program who answered at least one version of the COVID-19 Participant Experience (COPE) survey

We studied adults aged 40 and over who participated in the COVID-19 Participant Experience (COPE) survey, a nationwide survey administered by the NIH *All of Us* Research Program seeking to understand how the pandemic affected physical and mental health [20]. The exact survey instrument used can be found in the appendix. The survey is recurring, with six versions administered at the time of this study, beginning in May 2020 with the most recent iteration at the time of this study being February 2021. Participants were presented with the opportunity to answer one or more of these versions via e-mail. We obtained answers from each participant’s most recent version for our study. A total of 56,113 individuals were identified in the *All of Us* database who answered at least one version of the COPE survey, of which 42,484 (75.7%) were age 40 and over (Fig. 1). Glaucoma status was based on International Classification of Disease (ICD) diagnosis codes of any glaucoma type, including glaucoma suspect. Glaucoma suspect is a non-specific diagnosis used by clinicians to mean someone with a particularly high risk of glaucoma that requires close monitoring (may be high intraocular pressure, exam findings such as an enlarged cup to disc ratio, or a strong family history without a noted visual field deficit).

### Variables

We focused our analysis on demographics, mental health measures, and availability of social support if participants were infected with COVID-19. Demographic information was extracted from participants’ survey responses in the *All of Us* Basics survey [21]. Age in years was categorized as 40–64, 65–74, 75–84, ≥85. Racial and ethnic categories were coded as non-Hispanic (NH) White, NH Black/African American, NH Asian, and Hispanic (any race) individuals. Annual household income in dollars was categorized as 0-25 k, 25 k–50 k, 50 k–100 k, 100 k–200 k, > 200 k. Education was categorized as high school diploma/GED or lower, some college, and college and above. Insurance status was categorized as Medicaid, other insured (employer provided, privately purchased, Medicare, Military provided, VA provided, or other), and no insurance.

Mental health measures included calculation of Patient Health Questionaire-9 (PHQ-9) scores, a reliable measure of depression severity [22], where a score of 0–4 indicates none or minimal depression, 5–9 indicates mild depression, 10–14 indicates moderate depression, 15–19 indicates moderately severe depression, and ≥ 20 indicates severe depression. Because the PHQ-9 questionnaire was self-administered, we refer to depression as “probable depression.” We studied two different binary PHQ-9 cutoffs for probable depression, > 4 and > 9, as > 4 is a commonly used cutoff in clinical environments while > 9 has been shown to have high rates of sensitivity and specificity [22].

We also studied the following questions regarding stress and coping strategies experienced during the pandemic as follows: “Have recommendations for socially distancing caused stress for you?” and “To cope with social distancing and isolation, are you doing any of the following?” Measures of social support included whether participants had someone available to help if they were sick with COVID-19 and were confined to bed, and if someone was available to take them to the doctor, to help prepare meals, and to help with daily chores.

### Data analysis

Comparisons between various patient characteristics and survey responses were analyzed by glaucoma status with Pearson’s chi-squared tests to generate unadjusted *p*-values, using the Holm-Bonferroni adjustment for multiple comparisons which reduces the possibility of Type 1 error [23]. Assumptions for this non-parametric test were met, including comparing frequencies and not percents, comparing nominal or ordinal variables, comparing mutually exclusive levels within a variable, comparing independent study groups, participants falling into only one cell at a time, a value within cells ≥5 at least 80% of the time, and no cell < 1 [24]. We used logistic regression to generate odds ratios (ORs) and 95% confidence intervals (CIs) to characterize survey responses coded as binary (PHQ-9: severe/moderate/mild vs. minimal or no probable depression; stress from social distancing: a lot or some vs. a little or none; social support: always or most of the time vs. some, a little, or none of the time) by glaucoma status with non-glaucoma patients as the reference group. We calculated univariable and multivariable models.

Potential covariates for multivariable models were identified using a directed acyclic graph of known and suspected confounders for the association between glaucoma and mental health outcome [25]. Age, gender, race, ethnicity, education, income, and insurance status were considered. Paths between the exposure and outcome were identified using the back-door criterion [25]. We found that adjusting for age, gender, race, ethnicity, and income in the prior year provided the minimal sufficient adjustment. Statistical tests were two-sided, and *p*-values were considered statistically significant at the α = 0.05 level. Analyses were conducted on the NIH *All of Us* Researcher Workbench using R software version 4.1.0 and are available in the referenced notebook [26].

## Results

Of the 42,484 patients ages 40 and over who responded to *All of Us* COPE survey items, 2912 (6.9%) had a diagnosis of glaucoma. Most study participants were female (27,036, 63.6%), while 35,774 (84.2%) were NH White, 2523 (5.9%) were NH Black/African American, 907 (2.1%) were NH Asian, and 2343 (5.5%) were Hispanic (any race). Glaucoma patients had a median age of 72 years, with an interquartile range (IQR) from 66 to 77, while non-glaucoma patients had a median age of 67 years (IQR: 56–73). The most common income category was 50 k–100 k (12 971, 30.5%), and the majority (29,033, 68.3%) were college educated or above. The vast majority were insured 42,434 (99.9%), and of those 3446 (8.1%) had Medicaid insurance (Table 1).

**Table 1:** Demographic and socioeconomic characteristics among patients who answered the COVID-19 Participant Experience survey by glaucoma status

Characteristics^**a**^	Glaucoma	Non-Glaucoma	***P*** Value^**b**^
Total, No. (%)	2912 (6.9)	39,572 (93.1)	
Median age (IQR) in years	72 (65–77)	66 (56–73)	
Age category (in years), No. (%)			< 0.001
40–64	690 (23.7)	18,458 (46.6)	
65–74	1143 (39.3)	13,109 (33.1)	
75–84	963 (33.1)	7280 (18.4)	
> =85	116 (4.0)	725 (1.8)	
Gender, No. (%)			< 0.001
Female	1722 (59.1)	25,314 (64.0)	
Male	1190 (40.9)	14,258 (36.0)	
Race			< 0.001
White	2371 (81.4)	33,403 (84.4)	
Black/African American	241 (8.3)	2282 (5.8)	
Asian	72 (2.5)	835 (2.1)	
Other	190 (6.5)	2617 (6.6)	
Hispanic or Latino ethnicity			0.549
Yes	164 (5.6)	2179 (5.5)	
No	2683 (92.1)	36,637 (92.6)	
None of these	27 (0.9)	321 (0.8)	
Income (USD), No. (%)			
0-25 k	308 (10.6)	4100 (10.4)	< 0.001
25 k–50 k	506 (17.4)	6016 (15.2)	
50 k–100 k	952 (32.7)	12,019 (30.4)	
100 k–200 k	775 (26.6)	11,535 (29.1)	
> 200 k	295 (10.1)	5189 (13.1)	
Education, No. (%)			0.068
HS diploma/GED or lower	235 (8.1)	3128 (7.9)	
Some college	721 (24.8)	9158 (23.1)	
College and above	1941 (66.7)	27,092 (68.5)	
Health insurance, No. (%)			0.182
Other insured	2695 (92.5)	36,153 (91.4)	
Medicaid	199 (6.8)	3247 (8.2)	

*Abbreviations*: *NH* Non-Hispanic, *No*. Number, *IQR* Interquartile range, *HS* High school, *GED* General educational development

^a^Per the *All of Us* Research Program data sharing policies, cells with less than 20 respondents are suppressed and the number of missing responses are not included

^b^*P*-values were generated from Pearson’s Chi-squared tests using the Holm-Bonferroni adjustment for multiple comparisons

**Fig. 2:**
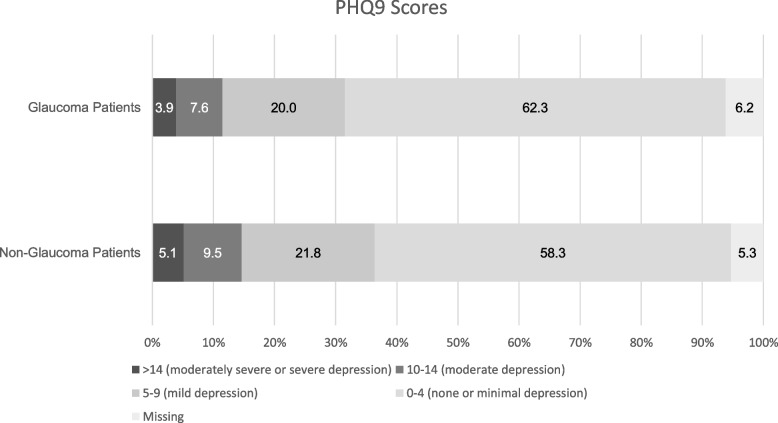
Bar chart showing Patient Health Questionaire-9 (PHQ-9) scores for patients who answered the COVID-19 Participant Experience (COPE) survey by glaucoma status

Over one-third of participants had a PHQ-9 score > 4 (glaucoma: 918, 31.5%; non-glaucoma: 14405, 36.4%), indicating mild, moderate, or severe probable depression (Fig. 2) – which significantly varied by glaucoma status (*p* = 0.001) on chi-square tests. Social distancing created some or a lot of stress for 765 (26.3%) glaucoma patients and 11,734 (29.7%) non-glaucoma patients, while 1139 (39.1%) glaucoma patients and 13,334 (33.7%) non-glaucoma patients reported not experiencing any stress at all – which also significantly varied by glaucoma status (*p* < 0.001). The most common coping strategies used while social distancing for both groups include engaging in behaviors such as eating healthy, getting exercise and plenty of sleep, and avoiding alcohol and drugs (glaucoma: 529, 18.2%; non-glaucoma: 6895, 17.4%). On Pearson’s Chi-squared tests glaucoma patients were less likely to report drinking alcohol (*P* = 0.003), eating more food than usual (*P* = 0.004), and using marijuana (*P* = 0.006) to cope with social distancing than those without a diagnosis of glaucoma (Table 2).

**Table 2:** Mental health and coping strategies used among patients who answered the COVID-19 Participant Experience Survey by glaucoma status

COPE Survey Questions^**a**^	Glaucoma	Non-Glaucoma	***P*** Value^**b**^
**PHQ9 Score**			< 0.001
0–4 (none or minimal depression)	1814 (62.3)	23,059 (58.3)	
5–9 (mild depression)	583 (20.0)	8607 (21.8)	
10–14 (moderate depression)	220 (7.6)	3774 (9.5)	
>14 (moderately severe or severe depression)	115(3.9)	2024 (5.1)	
NA	180 (6.2)	2108 (5.3)	
**Have recommendations for socially distancing caused stress for you?**			< 0.001
A lot	138 (4.7)	2657 (6.7)	
Somewhat	627 (21.5)	9077 (22.9)	
A little	950 (32.6)	14,112 (35.7)	
Not at all	1139 (39.1)	13,334 (33.7)	
NA	58 (2.0)	392 (1.0)	
**To cope with social distancing and isolation, are you doing any of the following?**			
Connecting with others, including talking with people you trust about your concerns and how you are feeling	241 (8.3)	3284 (8.3)	0.678
Contacting a healthcare provider	77 (2.6)	1012 (2.6)	0.746
Delaying medical care for conditions other than COVID-19	99 (3.4)	1234 (3.1)	0.229
Drinking alcohol	54 (1.9)	1126 (2.8)	**0.003**
Eating high fat or sugary foods	100 (3.4)	1458 (3.7)	0.181
Eating less food than usual	29 (1.0)	400 (1.0)	0.300
Eating more food than usual	109 (3.7)	1798 (4.5)	**0.004**
Engaging in healthy behaviors like trying to eat healthy, well-balanced meals, exercising regularly, getting plenty of sleep, or avoiding alcohol and drugs	529 (18.2)	6895 (17.4)	0.546
Increasing watching, reading, or listening to news stories, including social media	263 (9.0)	3412 (8.6)	0.925
Making time to relax	313 (10.7)	4132 (10.4)	0.752
Over exercise	<20	74 (0.2)	0.573
Smoking more cigarettes or vaping more	<20	243 (0.6)	0.499
Taking breaks from watching, reading, or listening to news stories, including social media	447 (15.4)	6556 (16.6)	0.200
Taking care of your body, such as taking deep breaths, stretching, or meditating	321 (11.0)	4093 (10.3)	0.773
Using cannabis or marijuana	<20	313 (0.8)	**0.006**
Using non-prescription drugs	<20	82 (0.2)	0.954
Using prescription drugs (like valium, etc)	<20	154 (0.4)	1.000
None of the above (if selected, no other response options are available	194 (6.7)	2600 (6.6)	0.257

^a^Per the *All of Us* Research Program data sharing policies, cells with less than 20 respondents are suppressed

^b^*P*-values were generated from Pearson’s Chi-squared tests using the Holm-Bonferroni adjustment for multiple comparisons

Between 40.5–51.3% of participants reported having social support all of the time if they were sick with COVID-19 and needed help. Glaucoma patients were less likely to report having someone to help prepare meals (*P* = 0.005) or help with daily chores (*P* = 0.003) if they became sick with COVID-19. However, glaucoma patients did not report significantly more difficulty with having someone to help if they were confined to bed (*p* = 0.144) or to take them to the doctor (*p* = 0.578) (Table 3).

**Table 3:** Social support measures among patients who answered the COVID-19 Participant Experience Survey by glaucoma status

COPE Survey Questions	Glaucoma	Non-Glaucoma	***P*** Value^**a**^
**Someone to help you if you were confined to bed**			0.144
All of the time	1199 (41.2)	16,632 (42.0)	
Most of the time	704 (24.2)	10,325 (26.1)	
Some of the time	393 (13.5)	5188 (13.1)	
A little of the time	266 (9.1)	3371 (8.5)	
None of the time	306 (10.5)	3620 (9.1)	
NA	44 (1.5)	436 (1.1)	
**Someone to take you to the doctor if you needed it**			0.578
All of the time	1467 (50.4)	20,314 (51.3)	
Most of the time	684 (23.5)	9575 (24.2)	
Some of the time	282 (9.7)	3916 (9.9)	
A little of the time	235 (8.1)	2916 (7.4)	
None of the time	200 (6.9)	2447 (6.2)	
NA	44 (1.5)	404 (1.0)	
**Someone to prepare your meals if you were unable to do it yourself**			0.005
All of the time	1278 (43.9)	17,974 (45.4)	
Most of the time	598 (20.5)	8971 (22.7)	
Some of the time	382 (13.1)	5051 (12.8)	
A little of the time	284 (9.8)	3418 (8.6)	
None of the time	311 (10.7)	3657 (9.2)	
NA	59 (2.0)	501 (1.3)	
**Someone to help with daily chores if you were sick**			0.003
All of the time	1179 (40.5)	16,415 (41.5)	
Most of the time	593 (20.4)	8962 (22.6)	
Some of the time	390 (13.4)	5438 (13.7)	
A little of the time	340 (11.7)	4138 (10.5)	
None of the time	340 (11.7)	3938 (10.0)	
NA	70 (2.4)	681 (1.7)	

^a^*P*-values were generated from Pearson’s Chi-squared tests using the Holm-Bonferroni adjustment for multiple comparisons

In univariable logistic regression, glaucoma patients were less likely than non-glaucoma patients to have a PHQ-9 score > 4 (OR: 0.80; 95% CI: 0.73–0.87), a PHQ-9 score > 9 (OR: 0.77; 95% CI: 0.67–0.87), to experience a lot or some stress from social distancing (OR: 0.83; 95% CI: 0.76–0.91), have someone help if they were confined to bed (OR: 0.89; 95% CI: 0.81–0.97),

and to have someone to help prepare meals if they were sick with COVID-19 (OR: 0.89; 95% CI: 0.81–0.97). In multivariable logistic regression adjusting for age, gender, race, ethnicity, and income, no difference was found for measures of mental health and social support between those with and without a diagnosis of glaucoma. (Table 4).

**Table 4:** Univariable and multivariable logistic regression for the association between measures of mental health and social support by glaucoma status, with non-glaucoma patients as the reference group

Logistic Regression^**a**^	Non-Glaucoma	Glaucoma
**Univariable**		
PHQ9 Score > 4 (mild/moderate/severe)	Ref	0.80 (0.73–0.87)
PHQ9 Score > 9 (moderate/severe)	Ref	0.77 (0.67–0.87)
A lot or some stress from socially distancing?	Ref	0.83 (0.76–0.91)
Someone to help you if you were confined to bed	Ref	0.89 (0.81–0.97)
Someone to take you to the doctor if you needed it	Ref	0.95 (0.86–1.04)
Someone to prepare your meals if you were unable to do it yourself	Ref	0.87 (0.80–0.95)
Someone to help with daily chores if you were sick	Ref	0.92 (0.84–1.00)
**Multivariable**^**b**^		
PHQ9 Score > 4 (mild/moderate/severe)	Ref	1.04 (0.95–1.14)
PHQ9 Score > 9 (moderate/severe)	Ref	1.05 (0.92–1.20)
A lot or some stress from socially distancing?	Ref	1.02 (0.93–1.12)
Someone to help you if you were confined to bed	Ref	0.92 (0.84–1.01)
Someone to take you to the doctor if you needed it	Ref	0.95 (0.86–1.05)
Someone to prepare your meals if you were unable to do it yourself	Ref	0.92 (0.84–1.01)
Someone to help with daily chores if you were sick	Ref	0.96 (0.87–1.04)

^a^Outcome variables were coded as binary as follows: PHQ9 (severe/moderate/mild vs. minimal or no depression); stress (a lot or some vs. a little or none); social support (always or most of the time vs. some, a little, or none of the time)

^b^Multivariable logistic regression adjusted for age, gender, race, ethnicity, and income

## Discussion

In this study of a nationwide cohort, we describe experiences on measures of mental health and social support during the early stages of the COVID-19 pandemic. Our cohort of glaucoma patients had a rate of mild, moderate, or severe probable depression (31.5%) that was over 1.7 times higher than what was reported in the general population in 2019 among those enrolled in the National Health Interview Survey (18.5%) [27]. Still, the glaucoma patients in our cohort had a lower unadjusted prevalence of probable depression than non-glaucoma patients, which is in line with some studies [28] and in contrast with others [12, 29–31]. This association was absent in multivariable logistic regression models, suggesting potential confounding by socioeconomic and demographic variables – particularly by race and age. The present study determined probable depression from self-reported questionnaires outside the context of a medical visit and thus were less prone to social desirability bias, which may explain why findings differ from previous studies. Furthermore, our study was based on survey responses gathered during the COVID-19 pandemic, when mental health concerns were highly prevalent across the general population.

How the COVID-19 pandemic differentially affected baseline depression rates among glaucoma patients relative to non-glaucoma patients is less clear and an area for future research. In any case, it is important for providers to be aware of their glaucoma patients’ mental health needs – not only because depression and anxiety make managing glaucoma difficult [29] but because there is evidence that anxiety is associated with faster retinal nerve fiber layer thinning, higher intraocular pressure, and disc hemorrhage [32], while depression is associated with visual field mean deviation [32] and an overall higher risk of developing glaucoma [33, 34]. This is part of a larger body of work that suggests that mental health can directly have systemic impacts through autonomic nervous system changes [35] (among other mechanisms) which is postulated to cause changes in blood flow and intraocular pressure that may accelerate glaucoma progression [32]. Though an early body of research, addressing mental health concerns appears increasingly relevant to providing comprehensive glaucoma care by having both social and potentially biological ramifications.

Nearly a third of participants endorsed feeling some or a lot of stress as a result of social distancing. Social isolation is a known cause of a variety of adverse mental health outcomes [36]. The most common methods both groups in this study utilized to cope with pandemic-related stress were activities that are generally healthy, including taking breaks from engaging with news and social media and engaging in exercise – which has been shown to promote well-being during the pandemic [37, 38]. Further, glaucoma patients were less likely to endorse maladaptive coping strategies such as drinking alcohol, overeating, or using marijuana. Though use of these maladaptive coping strategies appeared in relatively low numbers overall, they should be of concern to eye care providers as many are risk factors for disease progression [39].

Further, 77 (2.6%) glaucoma patients contacted a healthcare provider to help cope with social distancing and isolation, while 99 (3.4%) reported delaying medical care for conditions other than COVID-19. As glaucoma is a chronic progressive disease that is often asymptomatic, there is great concern over the effects of delaying care [6, 7]. This patient-driven delay in care likely contributes to the overall decrease observed in outpatient visits and surgical procedures in ophthalmology practices – a decrease which was more pronounced than any other medical specialty [40]. In addition, there is evidence that glaucoma medication adherence in the early stages of the pandemic decreased as well [41]. The health consequences of lower utilization have yet to be seen [7].

Lastly, over a third of participants reported having none, a little, or some help for various measures of social support if they were infected with COVID-19. On unadjusted Pearson’s Chi-squared tests, glaucoma patients were less likely to report having someone to help prepare meals or to help with daily chores if they became sick with COVID-19 than non-glaucoma patients. For other measures, there were still substantial proportions of glaucoma patients who endorsed not having social support all of the time (Table 3). Social support is often critical for glaucoma patients, as the disease primarily affects older individuals and may be associated with frequent follow-up visits, lifelong application of medications with sometimes complicated regimens, vision and autonomy loss, as well as accompanying expenses. Social support has been found to be positively correlated with quality of life and glaucoma treatment adherence [42–44]. One study found that low social support among glaucoma patients was significantly associated with increased rates of mental health disorders [45], and this in turn can further increase vulnerability to vision loss [29].

Social support among glaucoma patients during the COVID-19 pandemic has not been well characterized, but it is likely that pandemic conditions increased isolation and exacerbated any baseline lack of social support. In particular, glaucoma patients have been shown to have issues with transportation, which may make it difficult to obtain groceries [46] and is one of the most common reasons why glaucoma surgeries are canceled [47, 48]. Similarly, our study found that nearly half of patients with glaucoma did not always have someone to take them to the doctor if they needed it (Table 3). These transportation issues highlight the need for providers to offer a variety of access options, even in the years after the pandemic, including telehealth visits [5, 7], mobile eye clinics [49], and utilizing devices such as home-based intraocular pressure monitoring [50]. This is especially true for patients of low socioeconomic status, who already have lower disease awareness and treatment adherence at baseline [51].

Our study is not without limitations. First, we did not have data available for measures during the pre-pandemic period and were unable to quantify changes that may have been directly attributed to the COVID-19 pandemic. Second, as in many survey studies, responses may be influenced by social desirability bias. This is especially true with regards to coping strategies participants may or may have not partaken in to manage stressors associated with the pandemic. However, we do not expect social desirability bias to affect groups differently, and it likely has been at least partially mitigated in this anonymous/de-identified research survey compared to clinic-based studies where participants may be more concerned about how their physicians would perceive their responses. Third, we did not explore the effect of glaucoma severity, which has been shown to increase the likelihood of depression [13, 14] and possibly other metrics we studied. Fourth, there may have been some selection bias, as most of the *All of Us* participants who elected to participate in the COPE survey identified as White, were well-educated (> 90% had more than high school education), and all had some form of health insurance. This likely led to an under-estimation of mental health concerns and social stressors. Lastly, it must be noted that though many of these differences might be statistically significant, absolute differences are small for many measures and may not necessarily be clinically significant.

In conclusion, we report glaucoma patient experiences during the COVID-19 pandemic as compared to patients without glaucoma. A substantial proportion of glaucoma patients endorsed probable depression, social isolation, and difficulty with social support, even among a relatively well-educated and affluent population. Further, glaucoma patients may have had worse social support during the pandemic than patients without glaucoma. Still, much is unknown of the far-reaching effects of this pandemic on this patient population. Continued research is important moving forward as the possibility of new COVID variants and subsequent shutdowns may complicate efforts to return practice to normal. Further, an aging glaucoma population makes any mental health consequences related to this pandemic more relevant. Future research should seek to understand how the pandemic affected glaucoma patients through time as well as space (as lockdown measures varied considerably by region). Lastly, it is important to understand if any increases in depression or maladaptive coping strategies utilized by some patients will ultimately affect glaucoma outcomes. Aside from understanding these associations better, ophthalmologists should continue to address the less tangible factors that support eye health and vision outcomes – such as social determinants of health – and integrate patient social support structures into practice [44, 52].

## Data Availability

The NIH *All of Us* Research Program is a publicly available dataset. The link to the Researcher Workbench can be found here: https://www.researchallofus.org/data-tools/workbench/.

## Abbreviations

COVID-19: Coronavirus disease of 2019
NIH: National Institutes of Health
IRB: Institutional Review Board
UCSD: University of California San Diego
COPE: COVID-19 Participant Experience
ICD: International Classification of Disease
PHQ-9: Patient Health Questionaire-9
NH: Non-Hispanic

## References

[1] Pfefferbaum B, North CS (2020). Mental health and the Covid-19 pandemic. N Engl J Med.

[2] Bueno-Notivol J, Gracia-Garcia P, Olaya B, Lasheras I, Lopez-Anton R, Santabarbara J (2021). Prevalence of depression during the COVID-19 outbreak: a meta-analysis of community-based studies. Int J Clin Health Psychol.

[3] Collaborators C-MD. Global prevalence and burden of depressive and anxiety disorders in 204 countries and territories in 2020 due to the COVID-19 pandemic. Lancet. 2021;398(10312):1700-1712. 10.1016/S0140-6736(21)02143-7. 10.1016/S0140-6736(21)02143-7 PMC8500697 34634250

[4] Ettman CK, Abdalla SM, Cohen GH, Sampson L, Vivier PM, Galea S (2020). Prevalence of depression symptoms in US adults before and during the COVID-19 pandemic. JAMA Netw Open.

[5] Tan TE, Chodosh J, McLeod SD (2021). Global trends in ophthalmic practices in response to COVID-19. Ophthalmology..

[6] Holland LJ, Kirwan JF, Mercieca KJ. Effect of COVID-19 pandemic on glaucoma surgical practices in the UK. Br J Ophthalmol. 2022 106(10):1406–10. 10.1136/bjophthalmol-2021-319062. 10.1136/bjophthalmol-2021-319062 33931388

[7] Mahmoudinezhad G, Moghimi S, Weinreb RN (2020). COVID-19 pandemic: are we Back to Normal?. J Glaucoma.

[8] Dar S, De Moraes CG, Karani R (2021). Patient concerns regarding suspended ophthalmic care due to COVID-19. J Glaucoma.

[9] Zhang N, Wang J, Li Y, Jiang B (2021). Prevalence of primary open angle glaucoma in the last 20 years: a meta-analysis and systematic review. Sci Rep.

[10] Rao HL, Addepalli UK, Jonnadula GB, Kumbar T, Senthil S, Garudadri CS (2013). Relationship between intraocular pressure and rate of visual field progression in treated glaucoma. J Glaucoma.

[11] Grenard JL, Munjas BA, Adams JL (2011). Depression and medication adherence in the treatment of chronic diseases in the United States: a meta-analysis. J Gen Intern Med.

[12] Zhang X, Olson DJ, Le P, Lin FC, Fleischman D, Davis RM (2017). The association between Glaucoma, anxiety, and depression in a large population. Am J Ophthalmol.

[13] Mabuchi F, Yoshimura K, Kashiwagi K (2012). Risk factors for anxiety and depression in patients with glaucoma. Br J Ophthalmol.

[14] Skalicky S, Goldberg I (2008). Depression and quality of life in patients with glaucoma: a cross-sectional analysis using the geriatric depression Scale-15, assessment of function related to vision, and the Glaucoma quality of Life-15. J Glaucoma.

[15] Shweikh Y, Ko F, Chan MP (2015). Measures of socioeconomic status and self-reported glaucoma in the U.K. biobank cohort. Eye (Lond)..

[16] Zaher O, Ford RZ, Malvankar-Mehta MS (2022). Understanding the impact of COVID-19 on the quality of life of patients with eye disease. Exp Rev Ophthalmol.

[17] Pujari R, Chan G, Tapply I, Crc AG, Bourne RR (2022). The impacts of COVID-19 on glaucoma patient outcomes as assessed by POEM. Eye (Lond).

[18] Lesin Gacina D, Jandrokovic S, Marcinko D (2022). Anxiety and treatment adherence among Glaucoma patients during COVID-19 pandemic and earthquakes in Croatia. Psychiatr Danub.

[19] Denny JC, Rutter JL, All of Us Research Program I (2019). The "all of us" research program. N Engl J Med.

[20] NIH All of Us Research Program Investigators. COVID-19 Participant Experience (COPE). https://databrowser.researchallofus.org/survey/covid-19-participant-experience. Accessed 4 Nov 2021.

[21] NIH All of Us Research Program Investigators. Survey Explorer. https://www.researchallofus.org/data-tools/survey-explorer/healthcare-access-utilization-survey/. Accessed 5 Sept 2021.

[22] Kroenke K, Spitzer RL, Williams JB (2001). The PHQ-9: validity of a brief depression severity measure. J Gen Intern Med.

[23] Abdi H (2010). Holm’s sequential Bonferroni procedure. Encyclop Res Design.

[24] McHugh ML (2013). The chi-square test of independence. Biochem Med (Zagreb).

[25] Textor J, van der Zander B, Gilthorpe MS, Liskiewicz M, Ellison GT (2016). Robust causal inference using directed acyclic graphs: the R package 'dagitty'. Int J Epidemiol.

[26] Mental health and social support in the NIH all of us COVID-19 participant experience (COPE) survey - Analysis Notebook 2022. https://workbench.researchallofus.org/workspaces/aou-rw-4d3ba6a6/duplicateofsdhaineyeconditionsv5dataset/notebooks/preview/GlaucomaCOVIDv5Comparison.ipynb. 10.1186/s12886-023-02771-1 PMC9923653 36782129

[27] Villarroel MA, Terlizzi EP. Symptoms of depression among adults: United States, 2019. NCHS Data Brief. 2020;379:1–8. 33054920

[28] Wilson MR, Coleman AL, Yu F, Fong Sasaki I, Bing EG, Kim MH (2002). Depression in patients with glaucoma as measured by self-report surveys. Ophthalmology..

[29] Gamiochipi-Arjona JE, Azses-Halabe Y, Tolosa-Tort P (2021). Depression and medical treatment adherence in Mexican patients with Glaucoma. J Glaucoma.

[30] Mabuchi F, Yoshimura K, Kashiwagi K (2008). High prevalence of anxiety and depression in patients with primary open-angle glaucoma. J Glaucoma.

[31] Wang SY, Singh K, Lin SC (2012). Prevalence and predictors of depression among participants with glaucoma in a nationally representative population sample. Am J Ophthalmol.

[32] Shin DY, Jung KI, Park HYL, Park CK (2021). The effect of anxiety and depression on progression of glaucoma. Sci Rep.

[33] Jung Y, Han K, Wang SM, Yoon HY, Moon JI (2021). Effect of depressive symptom and depressive disorder on glaucoma incidence in elderly. Sci Rep.

[34] Berchuck S, Jammal A, Mukherjee S, Somers T, Medeiros FA (2021). Impact of anxiety and depression on progression to glaucoma among glaucoma suspects. Br J Ophthalmol.

[35] Bajko Z, Szekeres CC, Kovacs KR (2012). Anxiety, depression and autonomic nervous system dysfunction in hypertension. J Neurol Sci.

[36] Taylor HO, Taylor RJ, Nguyen AW, Chatters L (2018). Social isolation, depression, and psychological distress among older adults. J Aging Health.

[37] Kang HS, Kim BN (2021). The role of event-related rumination and perceived social support on psychological distress during the COVID-19 pandemic: results from greater Daegu region in South Korea. Psychiatry Investig.

[38] Ejiri M, Kawai H, Kera T (2021). Exercise as a coping strategy and its impact on the psychological well-being of Japanese community-dwelling older adults during the COVID-19 pandemic: a longitudinal study. Psychol Sport Exerc.

[39] Boland MV, Quigley HA (2007). Risk factors and open-angle glaucoma: classification and application. J Glaucoma.

[40] Poyser A, Deol SS, Osman L, et al. Impact of COVID-19 pandemic and lockdown on eye emergencies. Eur J Ophthalmol. 2021;31(6):2894–900. 10.1177/1120672120974944. 10.1177/1120672120974944 PMC8606945 33213198

[41] Racette L, Abu SL, Poleon S, Thomas T, Sabbagh N, Girkin CA. The impact of the COVID-19 pandemic on adherence to ocular hypotensive medication in patients with primary open-angle glaucoma. Ophthalmology. 2022;129(3):258–66. 10.1016/j.ophtha.2021.10.009. 10.1016/j.ophtha.2021.10.009 PMC8523310 34673098

[42] Stryker JE, Beck AD, Primo SA (2010). An exploratory study of factors influencing glaucoma treatment adherence. J Glaucoma.

[43] Wang Y, Zhao Y, Xie S, Wang X, Chen Q, Xia X (2019). Resilience mediates the relationship between social support and quality of life in patients with primary Glaucoma. Front Psychiatry.

[44] Budenz DL (2009). A clinician's guide to the assessment and management of nonadherence in glaucoma. Ophthalmology..

[45] Tilahun MM, Yibekal BT, Kerebih H, Ayele FA (2021). Prevalence of common mental disorders and associated factors among adults with Glaucoma attending University of Gondar comprehensive specialized hospital tertiary eye care and training center, northwest, Ethiopia 2020. PLoS One.

[46] Hochberg C, Maul E, Chan ES (2012). Association of vision loss in glaucoma and age-related macular degeneration with IADL disability. Invest Ophthalmol Vis Sci.

[47] Mehran N, Ojalvo I, Myers JS, Razeghinejad R, Lee D, Kolomeyer NN (2021). Surgical cancellations in Glaucoma practice: causes, delays, and effect on patient care and revenue. Ophthalmol Glaucoma..

[48] Hark LA, Radakrishnan A, Madhava M (2019). Awareness of ocular diagnosis, transportation means, and barriers to ophthalmology follow-up in the Philadelphia telemedicine Glaucoma detection and follow-up study. Soc Work Health Care.

[49] Hennein L, de Alba Campomanes AG (2021). Association of a Health Coaching and Transportation Assistance Intervention at a free ophthalmology homeless shelter clinic with follow-up rates. JAMA Ophthalmol.

[50] Mansouri K, Kersten-Gomez I, Hoffmann EM, Szurman P, Choritz L, Weinreb RN (2021). Intraocular pressure telemetry for managing Glaucoma during the COVID-19 pandemic. Ophthalmol Glaucoma.

[51] Hoevenaars JG, Schouten JS, van den Borne B, Beckers HJ, Webers CA (2006). Socioeconomic differences in glaucoma patients' knowledge, need for information and expectations of treatments. Acta Ophthalmol Scand.

[52] American Academy of Ophthalmology. 2022–2023 Basic and Clinical Science Course: Section 1, Update on General Medicine, Chapter 17 Social Determinates of Health. 2022. https://www.aao.org/Assets/af4d7f8f-10bd-4c85-a412-70f5e5de6fd1/637586691622930000/bcsc2022-s01-ch17-pdf.

